# Focused Screening of ECM-Selective Adhesion Peptides on Cellulose-Bound Peptide Microarrays

**DOI:** 10.3390/bioengineering3040031

**Published:** 2016-11-19

**Authors:** Kei Kanie, Yuto Kondo, Junki Owaki, Yurika Ikeda, Yuji Narita, Ryuji Kato, Hiroyuki Honda

**Affiliations:** 1 Department of Basic Medicinal Sciences, Graduate School of Pharmaceutical Sciences, Nagoya University, Aichi 464-8601, Japan; kanie-k@ps.nagoya-u.ac.jp (K.K.); yurika.ikeda.nagoya@gmail.com (Y.I.); kato-r@ps.nagoya-u.ac.jp (R.K.); 2 Department of Biotechnology, Graduate School of Engineering, Nagoya University, Aichi 464-8603, Japan; yuto.kondo.nagoya@gmail.com (Y.K.); junki.owaki.nagoya@gmail.com (J.O.); 3 Department of Cardiac Surgery, Nagoya University Graduate School of Medicine, Aichi 466-8550, Japan; ynarita@med.nagoya-u.ac.jp

**Keywords:** peptide microarrays, extracellular matrix, collagen type IV, clustering, amino acid index, physicochemical property

## Abstract

The coating of surfaces with bio-functional proteins is a promising strategy for the creation of highly biocompatible medical implants. Bio-functional proteins from the extracellular matrix (ECM) provide effective surface functions for controlling cellular behavior. We have previously screened bio-functional tripeptides for feasibility of mass production with the aim of identifying those that are medically useful, such as cell-selective peptides. In this work, we focused on the screening of tripeptides that selectively accumulate collagen type IV (Col IV), an ECM protein that accelerates the re-endothelialization of medical implants. A SPOT peptide microarray was selected for screening owing to its unique cellulose membrane platform, which can mimic fibrous scaffolds used in regenerative medicine. However, since the library size on the SPOT microarray was limited, physicochemical clustering was used to provide broader variation than that of random peptide selection. Using the custom focused microarray of 500 selected peptides, we assayed the relative binding rates of tripeptides to Col IV, collagen type I (Col I), and albumin. We discovered a cluster of Col IV-selective adhesion peptides that exhibit bio-safety with endothelial cells. The results from this study can be used to improve the screening of regeneration-enhancing peptides.

## 1. Introduction

In cases of long-term implantation of medical devices used to treat cardiovascular diseases, there is a critical risk of restenosis caused by thrombosis and neointimal hyperplasia [1]. Accelerated endothelialization is among the most effective means of preventing restenosis, thereby reducing the risks associated with vascular implants. Endothelialization involves reorganizing the damaged tissue around the implant area. The rapid and precise re-organization of vascular smooth muscle cells and fibroblasts is important for reducing the risk of neointimal hyperplasia. Accelerated re-endothelialization can be achieved on the surface of an implant using two different tissue-engineering approaches. The first involves seeding and culturing of endothelial cells (ECs) directly on implant surfaces, while the second involves enhancing the adhesion and growth of ECs through material surface design, frequently by using a bimolecular (protein or peptide) coating.

Several studies have reported effective medical implant design using biomolecules such as collagen [2], fibronectin [3], laminin [4], CD34 antibody [5], and extracellular matrix (ECM)-derived peptides [6,7,8,9] to mimic natural biological surface conditions. We have previously proposed designs for regeneration-enhancing medical implants and have reported several successful surface designs using trimer cell-selective peptides [10,11]. Using our original cell–peptide interaction screening method, a Peptide array–based Interaction Assay for Solid-bound Peptides, and an Anchorage-dependent Cell (PIASPAC) method [12,13], we have identified tripeptides that show selective adhesion, as determined by multiple assays comparing their relative affinities toward several cell types. Our tripeptides perform as well as other short, ECM-derived peptides, such as the REDV (Arg-Glu-Asp-Val) peptide derived from fibronectin [14] and the VAPG (Val-Ala-Pro-Gly) peptide derived from elastin [15].

The coating of medical devices with ECM-derived proteins, such as collagen type IV (Col IV), is an effective strategy for enhancing the endothelialization of device surfaces. However, ECM proteins such as Col IV, laminin, and elastin are difficult to produce using either recombinant or chemical synthesis approaches owing to the large sizes and hydrophobic properties of these molecules. Therefore, in order to enhance the performance of cell-selective peptides for faster regeneration, we sought to screen for peptides that can selectively accumulate target ECM proteins from blood vessels. Since Col IV is known to enhance endothelialization [16], in this study we screened for Col IV-selective adhesion peptides, i.e., peptides that selectively accumulate Col IV.

Supported by our previous success in finding cell-selective peptides, we chose the SPOT peptide microarray as our screening platform [17]. This platform exhibits several advantages in the screening of peptides for medical uses [14,15]. First, the screening platform is on a fibrous, three-dimensional surface. As shown in our previous work, artificial blood vessels, particularly those expected to be used in regenerative medicine, are made of fiber materials that enhance cellular growth and mobility for tissue regeneration. Since it is known that the topological surface greatly influences cellular adhesion/migration behavior, flat supports such as glass are not ideal for screening in spite of their ability to house a large peptide library. Therefore, it is better to evaluate the function of peptides on a platform with a biologically relevant surface. The three-dimensional fibrous structure is also important for increasing the signal-to-noise ratio in array-type screens when probes are limited or expensive. In order to expand peptide screening for various ECM proteins, this aspect was considered critical. It is also known that fiber-derived three-dimensional structure arrays, such as CelluSpotsTM, provide higher peptide densities in the spot area, allowing for the detection of weak interactions. Another advantage is the extreme hydrophilicity of cellulose membranes, which are known to show low protein adhesion, resulting in a reduction in non-specific binding of proteins. Owing to these properties, SPOT peptide microarrays have exhibited high performance in epitope mapping with low background noise.

**Figure 1:**
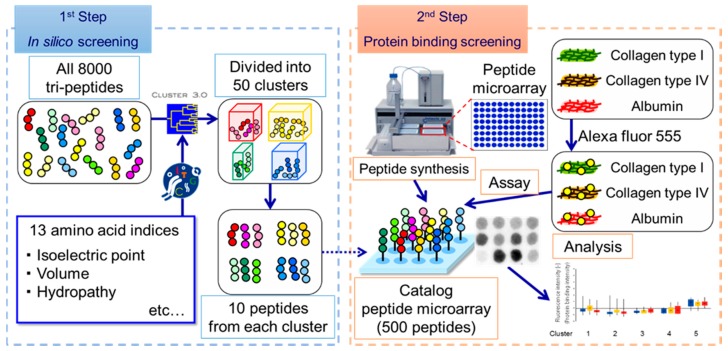
Schematic illustration of our concept for screening for ECM-selective (extracellular matrix) adhesion peptides. The method is divided into two sequential steps. The first step involves in silico screening of tripeptides. Peptide sequences are categorized according to 13 amino acid indices and are clustered in silico into 50 groups of physicochemically similar peptides. Ten representative peptides are then selected from each cluster. In the second step, these representative sequences are synthesized on the candidate array. In our assay for detecting ECM-selective peptides, microarray intensities indicate the normalized, relative strengths of protein adhesion to Col IV (collagen type), Col I, and Alb. Finally, the results are analyzed to identify Col IV-selective adhesion peptides.

Despite these advantages, the largest disadvantage of the SPOT peptide microarray in terms of screening for ECM-selective peptides was the limited size of the library. Therefore, in this study, we introduced a clustering-assisted focused screening method, which has been demonstrated to enhance screening [13]. The essence of this screening concept is to minimize the risk of redundantly including highly similar peptides on the limited area of the custom array. In this method, by first clustering physicochemical properties of peptides in silico, physicochemically diverse types of peptides determined to be representative are synthesized on an array to screen for properties that satisfy the target function (Figure 1). We chose to screen trimer peptides for the discovery of Col IV-selective adhesion peptides for three reasons. First, it is our hope that these peptides will eventually be used in medical device coatings. For medical usage, peptides must be synthesized and purified at a commercial scale. As peptide length increases, synthesis efficiency can drop drastically and the purification process can suffer with hydrophobic sequences. Moreover, with present fermentation technology, tripeptides can be produced biologically at a lower cost and with less liquid waste. Second, trimer peptides are free from structural conformations. With peptides longer than 5-mers, there is the possibility of forming helices. If such conformations form on peptide microarrays, interpreting the assay results becomes extremely difficult. Lastly, we plan to combine the target Col IV-selective adhesion peptides with previously obtained cell-selective peptides to mimic ECMs. For this purpose, it was necessary to use the same trimer peptides as in our previous work.

In this report, we were able to successfully implement this screening concept to identify a cluster of adhesion trimer peptides that selectively bind to the target ECM protein Col IV over the non-target ECM proteins collagen type I (the major collagen found in vivo) and albumin (the major ECM protein in serum). Moreover, these Col IV-selective adhesion peptides were shown to exhibit bio-safety with endothelial cells. 

## 2. Materials and Methods 

### 2.1. Design of Custom SPOT Peptide Microarray

**Figure 2:**
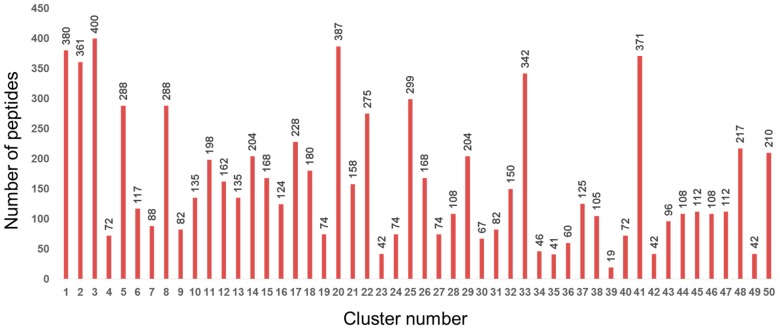
Number of peptides in each of the 50 clusters containing physicochemically similar peptides.

We first clustered 8000 tripeptide sequences (covering every sequential combination) in silico (Figure 1). The physicochemical features of each peptide were calculated by considering each amino acid in order from the N-terminus. Each sequence of amino acids was characterized according to 13 indices (Table 1) selected from AAindex1 as reported on Genome Net Japan, which is organized by Kyoto University [18,19]. The tripeptide profile was then converted into 39 features (13 indices × 3 sequential positions). Using the feature data, average-linkage hierarchical clustering was implemented by Cluster 3.0, distributed by Michiel de Hoon et al. from the University of Tokyo’s Human Genome Center, Tokyo, Japan [20]. The results of hierarchical clustering were visualized with the open source tool Maple Tree version 0.2.3.2 BETA (Lawerence Berkeley National Laboratory., Berkeley, CA, USA) [21]. Clustering revealed 50 clusters of physicochemically similar peptide sequences (Figure 2). From each cluster, ten representative peptides were randomly selected, resulting in a set of 500 candidate peptides that should encompass the maximum diversity of physicochemical properties (Figure 1).

Using the library of representative peptides, a custom SPOT peptide microarray was synthesized by F-moc chemistry following a previous report [10] with modifications. To ensure reproducibility, we produced three arrays for the same library with each peptide included in triplicate and all spots randomly positioned on the three arrays to reduce positional bias. As internal controls, 20 peptides (AAA, CCC, DDD, EEE, FFF, GGG, HHH, III, KKK, LLL, MMM, NNN, PPP, QQQ, RRR, SSS, TTT, VVV, WWW, and YYY) were spotted on each array.

### 2.2. Protein Accumulation Assay on Peptide Microarray

Human collagen type I (Col I; Santa Cruz Biotechnology, Dallas, TX, USA), human collagen type IV (Col IV; Collagen Research Center, Tokyo, Japan), and human serum albumin (Alb; MP Biomedicals, Newport Beach, CA, USA) were labeled with Alexa Fluor 555 succinimidyl ester (Invitrogen, Carlsbad, CA, USA). Peptide microarrays were hybridized with 20 ng/ml labeled protein for 2 h for each protein in turn. Peptide spots were scanned with a Typhoon FLA-7000 laser scanner (Fujifilm, Tokyo, Japan) at 532 nm/585 nm (excitation/emission) wavelengths. Recorded fluorescence intensities for all three proteins were analyzed with ArrayGauge Ver.2.1 (Fujifilm, Tokyo, Japan). Intensities were summarized, normalized, and analyzed by a previously reported method with slight modifications [33]. 

### 2.3. Cell Culture

Normal human umbilical vein ECs (Kurabo Industries, Osaka, Japan) were cultured in HuMedia-EG2 medium (Kurabo Industries, Osaka, Japan) at 37 °C under 5% CO_2_. Cells from passages 4–6 were used.

### 2.4. Scanning Electron Microscopy

Cells were treated according to the cell assay protocol described for the PIASPAC method [12,13]. Cells growing on peptide-coated disks were fixed in 4% glutaraldehyde (Wako Pure Chemical Industries, Tokyo, Japan) for 12 h at 4 °C. Following a second fixation step using osmium tetroxide (PGM Chemicals (Pty), New Germany, RSA) for 30 min at 28 °C, samples were dried with t-butylalcohol (Wako Pure Chemical Industries, Osaka, Japan) and a VFD-20 drying apparatus (Hitachi, Tokyo, Japan). Samples were subsequently coated with osmium tetroxide using a plasma coater (Nihon Lazer Denshi, Ichinomiya, Japan). Scanning electron microscopy (SEM) images were obtained with an S-800 electron microscope (Hitachi, Tokyo, Japan).

### 2.5. Cell Adhesion Assay

A cell adhesion assay was conducted on SPOT arrays according to a previously described method [13] with slight modifications. Each spot from the synthesized peptide array, corresponding to a different peptide, was punched out as a disk and embedded in a 96-well plate. Cells were stained with calcein AM (Life Technologies Corporation, Carlsbad, CA, USA) for 30 min, and 1.5 × 10^4^ cells/well were directly seeded on the disks with appropriate cell culture medium. Cells and peptide disks were incubated for 1 h for cell adhesion. After three washes of phosphate-buffered saline by pipetting to remove unattached cells, fluorescence intensity was measured on a Fluoroskan Ascent (type 374; Labsystems, Helsinki, Finland) at 485/538 nm (excitation/emission) wavelengths. For reproducibility, data from triplicate spots were averaged.

## 3. Results and Discussion

### 3.1. Screening of Col IV-Selective Adhesion Peptides

**Figure 3:**
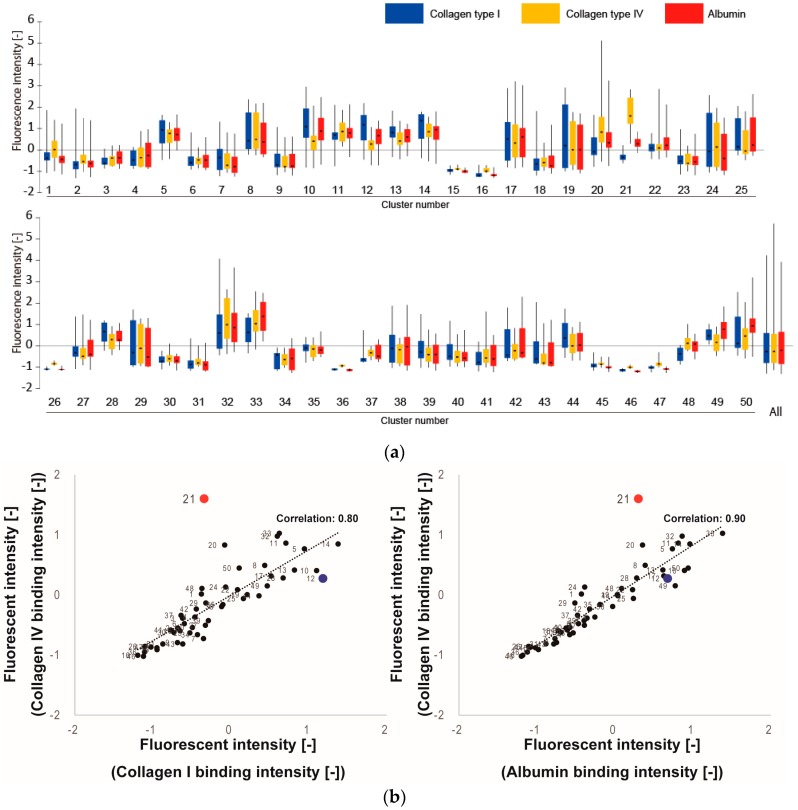

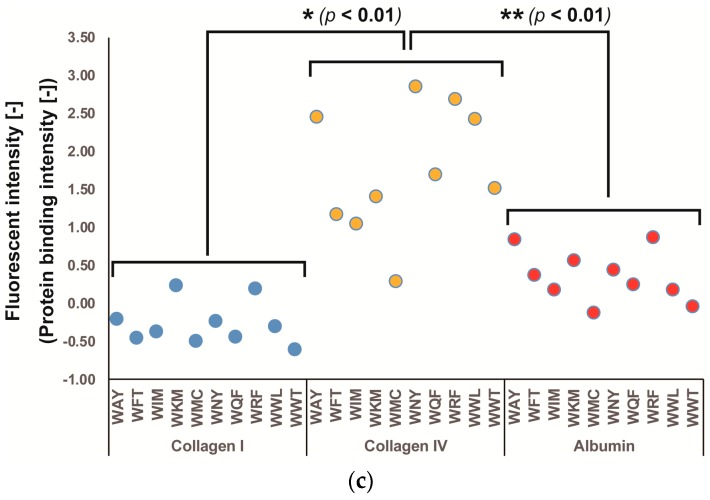
Results of screening for ECM-selective peptides. (**a**) The average fluorescence intensities of triplicate peptide spots are summarized for all clusters with box plots. The label “All” refers to the summarized value of all 500 tripeptides. For each cluster, the median value of the ten peptides is shown as a horizontal line, the 50% quantile area is shown as a box, and the standard deviation is shown as whiskers; (**b**) Correlation between Col IV and Col I or Alb in 50 clusters. Numeric character indicates cluster number. Red dots: cluster 21, blue dots: cluster 12; (**c**) Detailed plot of peptide binding rates in cluster 21 (Col IV-selective adhesion cluster). Each plot is representative of nine data points (triplicate spots from three different arrays). Fluorescence intensity values were normalized by standard normalization (average = 0, standard deviations = 1). * Denotes statistical significance compared to collagen I, *p* < 0.01, Student’s *t*-test. ** Denotes statistical significance compared to albumin, *p* < 0.01, Student’s *t*-test.

During screening, we compared the binding rates and relative selectivities of the representative peptides to three ECM proteins: Col IV (target ECM protein that could control ECs), Col I (ubiquitous, non-target ECM protein that could adhere any types of cells), and Alb (major non-target protein in the blood with no role in controlling cells). Figure 3a shows the measured fluorescence intensities for each of the 50 clusters. In each cluster, most representative peptides exhibited similar binding properties compared to the total standard deviation across all 500 peptides. However, since the standard deviations within each cluster were larger than anticipated, we concluded that 50 clusters were not sufficient to produce homogeneous clusters in the categorization of ECM-tripeptide interactions. However, we did identify cluster 21 as exhibiting higher signal intensity in binding to Col IV and lower intensities in binding to Col I and Alb (Figure 3a). To indicate the reason why cluster 21 was selected, correlation analysis were performed between Col IV and Col I, Col IV, and Alb (Figure 3b). Additionally, the correlation score increase when cluster 21 is excluded (Col IV vs Col I: from 0.80 to 0.88, Col IV vs Alb: from 0.90 to 0.94). From the results, cluster 21 is different than the other clusters. Interestingly, neither Col I-selective nor Alb-selective clusters were found. Within cluster 21, the tripeptides WNY, WRF, WAY, and WWL exhibited high binding selectivity (Figure 3c) for Col IV. Since cluster 21 is not a large cluster (2% of 8000 peptides), the fact that we identified Col IV-selective adhesion peptides in the first screen indicates that our clustering-assisted library design did indeed enhance screening efficiency.

There are several collagen binding peptides that have been discovered from von Willebrand factor (vWF) or CNA35, M-like surface proteins, and osteopontin (OPN) in many research studies [34,35,36]. The WREPSFCALS peptide derived from vWF has been reported to bind bovine collagen I [37]. The octa-peptide motif from different streptococcal species, AXYLZZLN, is present in M-like surface proteins which bind human Col IV [38]. The GLRSKSKKFRRPDIQYPDATDEDITSHM peptide was identified from the residues 150–177 of human OPN [39]. Moreover, the HVWMQAP peptide was discovered by using a phage display approach [40]. 

Comparing other collagen binding peptides, this study’s Col IV-selective adhesion peptides have some advantages. This study’s peptides are short trimer peptides and have Col IV selectivity. Thus the sequence obtained from this study was different from these previous collage binding peptides. However, the variety of amino acid constructing previous peptides is only slightly similar to the peptides discovered from this study. The common points are the few positively charged amino acids such as R and K (1.7% in cluster 21), and many hydrophilic amino acids such as V, L, and M (6.5% in cluster 21), and many aromatic amino acids such as F, Y and W (6.1%–36.7% in cluster 21).

### 3.2. Physicochemical Properties of Col IV-Selective Adhesion Peptides

**Figure 4:**
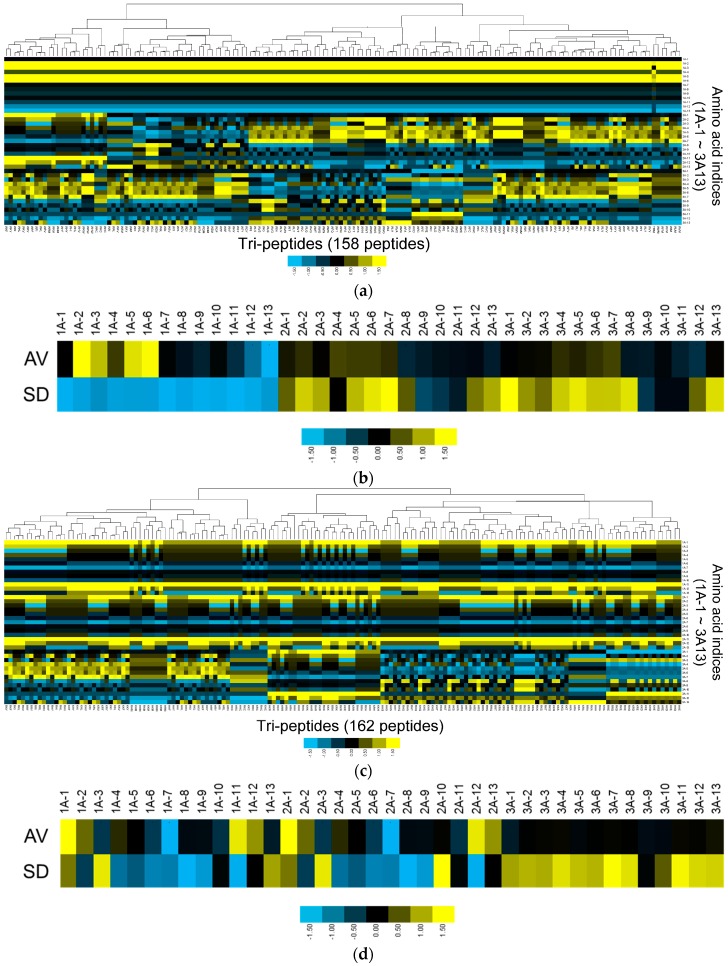
Physicochemical property analysis for cluster 21 (Col IV-selective adhesion peptides) and cluster 12 (Col I-selective adhesion peptides). (**a**) Heat map showing patterns of amino acid indices at each residue in cluster 21; (**b**) Averages and standard deviations of amino acid indices at each residue within cluster 21; (**c**) Heat map showing patterns of amino acid indices at each residue in cluster 12; (**d**) Averages and standard deviations of amino acid indices at each residue within cluster 12. Amino acid indices are designated as “position-index number”. For example, 1A-1 represents index 1 at the first amino acid (1A) in the tripeptide.

In order to investigate the physicochemical properties of the Col IV-selective adhesion peptide cluster, we analyzed the amino acid indices from cluster 21 in detail. We compared these to the amino acid indices from cluster 12, which exhibited relatively high binding to Col I rather than Col IV. Heat maps of the physicochemical properties of the peptides in these two clusters (Figure 4) reveal obvious differences. In particular, the patterns for amino acid indices 1, 2, 3, 5, 6, 11, 12, and 13 at the first (N-terminal) residue and those for amino acid indices 1, 7, 11, and 13 at the second residue differed substantially between cluster 21 and cluster 12. Conversely, patterns of amino acid indices for the third (C-terminal) residue were similar in both clusters. We conclude that the physicochemical properties that confer Col IV-selective adhesion properties to cluster 21 are driven by these amino acid index patterns. 

Figure 4b,d show the averages and standard deviations (SD) of the amino acid indices in each cluster at each position. At the N-terminal residue, cluster 21 exhibits a homogeneous pattern that differs considerably from that of cluster 12. Therefore, although the overall heat map reveals that there are still several smaller clusters of peptides included in cluster 21 (Figure 4a), the cluster’s overall propensity for Col IV-selectivity may be represented by the N-terminal residue.

### 3.3. Bio-Safety of Col IV-Selective Adhesion Peptides with ECs

In a previous study, we discovered an EC-selective peptide that exhibits better selective adhesion than RGDS (Arg-Gly-Asp-Ser), a strong cell adhesion peptide that binds to integrin αvβ3. Our focus in this screen, therefore, was to discover a peptide that will support regeneration by accumulating ECM proteins from the blood. With this concept in mind, it is important to consider the bio-safety (i.e., the presence of absence of cell toxicity) of the Col IV-selective adhesion peptides with ECs. From past studies, we know that some tripeptides are able to eliminate EC adhesion [10,33].

**Figure 5:**
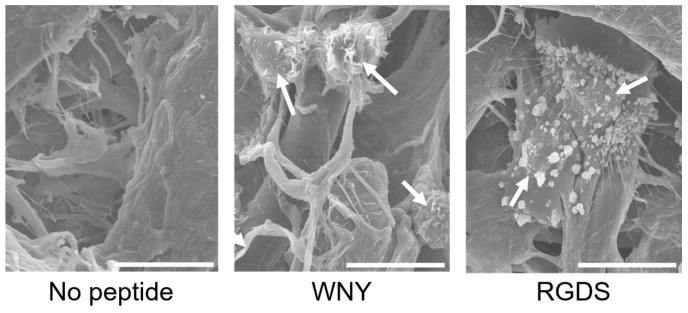
Scanning electron micrograph images of endothelial cells (ECs) on a Col IV-selective adhesion peptide (WNY)-coated and RGDS peptide-coated cellulose disk after 1 h of incubation. Adhered cells remaining after the washing procedure are indicated with white arrows. Scale bar, 15 μm.

**Figure 6:**
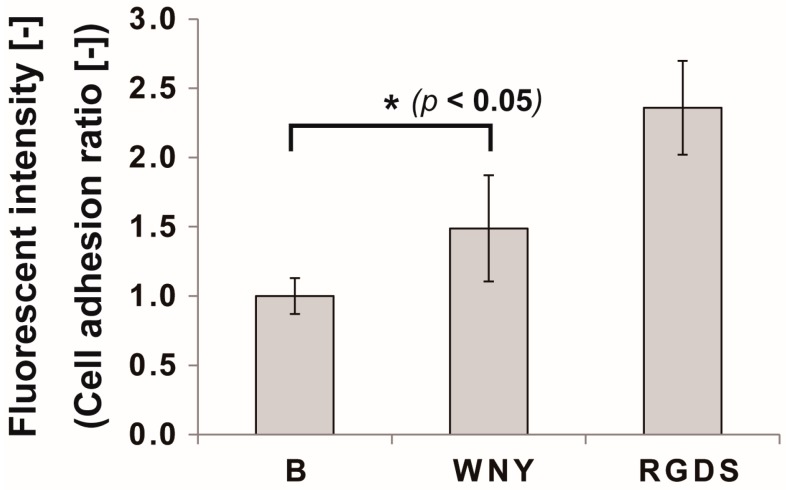
Results of the cell adhesion assay with cellulose disks coated in Col IV-selective adhesion peptide (WNY) or RGDS peptide. Cell numbers were measured by CalceinAM staining of live cells after 1 h of incubation. B: blank disk with no peptide coating. All experiments were performed six times (* *p* < 0.05, Student’s *t*-test).

Therefore, we investigated the effect of a Col IV-selective adhesion peptide coating on an EC culture (Figure 5). We selected the WNY peptide from cluster 21 for this experiment, as it was the top Col IV-selective adhesion peptide based on the results of our screen (Figure 3c). When ECs were cultured with the WNY peptide-coated cellulose disk for one hour, cells were found to adhere to the fibers (Figure 5). Adhesion morphology, however, differed from that of ECs incubated with the RGDS peptide. Cells exhibited a more flat morphology with more filopodia when incubated with RGDS. On the other hand, cells on the WNY peptide exhibited a round shape with a few filopodia but adhered well as opposed to on no peptide. This is because that cell can adhere by “physicochemical properties” interactions, not “ligand-receptor” interactions as described in our previous study [10,12]. By counting the total number of cells on the peptide disk, we found that the total cell adhesion rate was 1.5-fold higher on the WNY-coated membrane than that of a blank cellulose membrane (Figure 6). However, the WNY-coated membrane did not elicit as strong a cell adhesion effect as the RGDS-coated membrane. Nevertheless, WNY represents a candidate peptide that should be further investigated in combination with our previously obtained cell-selective peptides [10], for accelerating re-endothelialization.

## 4. Conclusions

In this study, we presented the results of a screen for Col IV-selective adhesion peptides using SPOT peptide microarrays. SPOT peptide microarrays offer many advantages for the screening of peptides in solid-bound form on fibrous scaffolds for regenerative medicine. The library size limitation, however, had to be overcome in order to conduct an effective screen. For this, we introduced a clustering-assisted library design concept from our previous work, as ECM-selective adhesion tripeptides have not been previously screened and could not be determined by property-based investigation. Through this screening protocol, we identified a cluster of Col IV-selective adhesion peptides. Further analysis of this physicochemical cluster suggested that the N-terminal amino acid in the trimer peptide contributes strongly to Col IV-selective binding. Although we may have identified rare peptides exhibiting the target binding properties without the in silico clustering regime, clustering provided us with additional information we used to better understand the binding properties of these peptides. This feedback loop of experimental and informatics analyses will therefore allow us to refine the efficiency and accuracy of peptide design [13]. Our next step is to further evaluate the efficacy of our screened peptides in vitro and in vivo by determining the hybridization kinetics of peptide binding, as well as the effects of probe-labeled fluorophores and other blood components during a second, more comprehensive round of screening. Moreover, our final goal is to design biomaterials that accelerate re-endothelialization. Thus it is necessary to design the biomaterials that will be synthetic polymer or biopolymer immobilized with peptides that we discovered in this or previous studies. The results from this study can be used to improve the screening of regeneration-enhancing peptides.

## Figures and Tables

**Table 1:** The 13 amino acid indices (selected from a total of 531 indices) used for characterization of tripeptides.

Index Number	Description	Reference
1	Isoelectric Point	[22]
2	Normalized van der Waals Volume	[23]
3	Alpha-Helix Indices for Beta-Proteins	[24]
4	Beta-Strand Indices for Beta-Proteins	[24]
5	Side-Chain Contribution To Protein Stability	[25]
6	The Stability Scale from the Knowledge-Based Atom–Atom Potential	[26]
7	Hydropathy Index	[27]
8	Normalized Frequency of Turn	[28]
9	Free Energy in Beta-Strand Region	[29]
10	Free Energy in Alpha-Helical Region	[29]
11	Polarity	[30]
12	Side Chain Interaction Parameter	[31]
13	Amino Acid Distribution	[32]
